# Costs of air pollution impact on health in the Western Balkans: preliminary results

**DOI:** 10.1093/eurpub/ckac129.144

**Published:** 2022-10-25

**Authors:** C Belis, M Ballocci, V Matkovic, G Millo, M Jevtic, R Van Dingenen

**Affiliations:** 1 European Commission, Joint Research Centre, Ispra, Italy; 2 Health & Environment Alliance HEAL, Brussels, Belgium; 3 DEAMS, University of Trieste, Trieste, Italy; 4 Faculty of Medicine, University of Novi Sad, Novi Sad, Serbia; 5 Institute of Public Health of Vojvodina, Novi Sad, Serbia

## Abstract

Air pollution is the main environmental driver associated with health. It is well documented that poor air quality is responsible for increased risk of mortality and morbidity. The social cost of mortality in 2015 was estimated in 3 trillion (OECD, 2016). The Western Balkans (WB) comprise Albania, Bosnia and Herzegovina, Kosovo*, North Macedonia, Montenegro and Serbia covering area of 218 750 km2 and a population of 19.9 million with total GDP of €94.2 billion (Banja et al., 2020). The WB was selected for this study because it is one of the air pollution hotspots in Europe where the levels of PM2.5, PM10, NO2 and O3 are frequently above the EU Air Quality Directive guidelines. This situation has been associated with a higher proportion of premature deaths attributable to air pollution exposure (4-19% of total deaths) in this region compared to EU member states (EEA, 2021). The health impacts including mortality and morbidity were estimated for particulate matter PM2.5, ozone (O3) and nitrogen dioxide (NO2) at country and city level on the basis of exposure in 2019 derived from monitoring stations and model estimations. Mortality impacts were parameterised using the number of premature deaths. Morbidity costs included: chronic bronchitis, hospital admissions due to respiratory diseases, hospital admissions due to cardiovascular diseases, bronchitis in children, asthma in children, reduced activity days and work lost days. The costs of mortality attributable to air pollution were estimated on the basis of non-market welfare based methods (WTP approach) while morbidity costs were estimated mainly with market based methods combining both direct and indirect costs. The 2019 health costs, both per capita and as share of the GDP, associated with air pollution in the WB were considerably higher than those in EU27.

